# Industrial washing conditions as factor that influence the cellulose structure and mechanical strength of bed linens

**DOI:** 10.1038/s41598-023-38969-y

**Published:** 2023-07-27

**Authors:** Izabela Jasińska

**Affiliations:** Lukasiewicz Research Network, Lodz Institute of Technology, 19/27 M. Sklodowskiej-Curie, 90-570 Lodz, Poland

**Keywords:** Mechanical properties, Chemical engineering

## Abstract

It is crucial for companies providing rental and maintenance services to hotels and hospitals to tackle the problem of decreased physical longevity caused by frequent laundering procedures in the industrial textile sector. Proper maintenance of bed linens is vital as they undergo multiple rigorous preservation techniques, such as being treated with chlorine to remove tough stains and sanitize the fabrics. The mechanical strength of fabric made of 100% cotton fibre products greatly relies on the degree of polymerization of cellulose-cotton fibre material. The study evaluated the washing performance of five cotton fabrics. Two weaving pattern variations were used and the fabrics were composed either of 100% cotton or a blend of 50% cotton and 50% polyester fibres. The washing methods included commercial and chlorine-based laboratory washing. 100% cotton fabrics, especially in plain weave show higher tensile strength falls then blended ones. The pure cotton fabric loss much more of its initial strength after only few chlorine-based washings than after hundred commercial ones. Limiting viscosity number values drop in half after hundred commercial washings for cotton fibres taken from tested woven fabrics. In comparison, decline of this parameter after only ten chlorine-based washings is more than 80% of their initial values. Performing the maintenance process without free chlorine, while still retaining its high effectiveness, can notably augment the frequency of maintenance procedures and preserve the mechanical durability of cotton fabrics over a longer time span. This leads to a reduction in textile waste residues.

## Introduction

Repeated industrial laundering processes can cause a loss of mechanical strength in textile products. This is a significant issue for companies that offer rental and maintenance services for items such as bed clothes. Bed linens utilized in hotels or hospitals undergo numerous preservation procedures, frequently in severe circumstances, such as the use of free chlorine treatment to eliminate tough stains and sanitize the items. In the case of 100% cotton fibre products, degree of polymerization of cellulose-cotton fibre material, plays a major role. Damage to the glucoside bonds of the cellulose macromolecule chain occurs mainly as a result of low pH factors and to a lesser extent alkalis. In oxidizing environments, particularly at neutral pH, changes in the degree of polymerization do not directly reflect the level of loss of mechanical strength. The assessment of variations in the polymerization degree of cellulose is extremely important for determining the strength and longevity of paper products^1^. Moreover, it plays a crucial part in producing ground breaking nanostructures that are extracted from cellulose obtained from leftover palm fruit, specifically waste from empty oil palm fruit bunches^2^. In the 1980s, research was conducted on the effects of washing on the mechanical properties of cotton fibres and the spinning process. The study focused on the influence of washing on these factors. Several papers were published on this research during that time period^3–6^. The influence of chlorine based treatment used mainly for removing persistent stains and maintain cotton fabric’ whiteness were investigated since 1940s^7,8^. The results presented by researchers shown significant decrease in both properties for fabrics after bleaching and alkaline treatment.

A recent study conducted by experts at the University of Prague^9^ has concluded that the best approaches to measure cellulose degradation in textile products are by analysing the degree of polymerization and evaluating the tensile strength of yarns. The paper investigated the usefulness of spectroscopic techniques, such as FTIR and UV–Vis. These techniques have significant limitations in evaluating β 1–4 glycosidic bond degradation or glucopyranose ring opening in cellulose macromolecules, as per the analysis results. The study described in paper^10^ investigates the impact of repeated laundering, whether at home or in an industrial setting, on the alteration of the degree of polymerization exhibited by cotton, viscose, and blended cellulose fabrics. This includes hospital bed linens as well. The results indicate that cotton fabrics experienced the most significant reduction in polymerization degree after multiple washing and drying cycles. This reduction amounted to 15% for household washing and even up to 80% for industrial washing.

The quality of specific products such as hospital bedclothes, towels, pyjamas, medical personnel clothing (surgeons), hotel bedclothes, and work clothes were analysed by researchers from the Universities of Maribor and Zagreb. They conducted a study on the impact of their team’s industrial washing procedures^11^. The study focused on investigating washing procedures of a model fabric based on DIN 53919-1:1980/ISO 2267 standards. Both the RAL guidelines within RAL-GZ 992 and the EMPA-created document provided specifications for the allowable variations in mechanical strength, polymerization degree, ash content, and whiteness degree.

The researchers have determined that the developed washing procedures are optimal for meeting the requirements of the aforementioned documents. However, it is necessary to further analyse their effectiveness in removing contaminants such as microbiological and heavy soiling.

Similar works were done by Pusic and their team^12^, where industrial washing conditions dedicated to linen from kitchen, restaurant and hotel were investigated. The mechanical strength of fabric, depolymerization of cotton fibres, as well as colour changes due to fading or dye transfer were carefully examined by researchers. They also analysed greying and alterations in shade. Moreover, surface modification, which occurred during the washing cycles, was monitored and evaluated by streaming potential and scanning microscopy. Another issue is the effect of finishing treatment of cotton textile products on their mechanical durability. Publication^13^ investigates the impact of anti-creasing finish on the mechanical strength of 100% cotton fabrics. Although the researchers were able to successfully achieve the anti-creasing effect and shrinkage reduction, they did observe a noteworthy decrease in the strength of the fabrics. Finishing treatments and frequent washing or cleaning—especially with water—are two major culprits for the reduced mechanical durability of fabrics. In fact, water washing can cause a strength loss of up to 30% compared to unfinished fabric. The washing process analysis must also consider the assessment of the mechanical effects on washed items, which originate from the structural components of the machine and the washing settings. The works^14,15^ of researchers from Seoul National University (Korea) showed that each of the above-mentioned factors influences the quality of the washing process.

Fabrics used for hotel or hospital bed clothes undergo intense maintenance processes. Therefore, they must meet specific requirements to ensure the processes do not cause excessive damage to them. Requirements in this file were elaborated by the German Certification Association for Professional Textile Services together with Institute Hohenstein^16^. Similar requirements and certification process are offered by Textile Testing Institute (TZU) from Brno, Czech Republic^17^. One crucial parameter is the highest tolerable reduction in fabric strength following repeated industrial washing, set at 30% compared to the fabric’s initial strength^18,19^. Moreover, standard^19^ outlines the prerequisites for bedding products that are meant for everyday usage. Table 1 displays the minimum maximum strengths acceptable for fabrics as well as the maximum permitted alteration in dimensions after three washings and drying at 90 °C. Low-quality bed linens provided in hotels or hospitals quickly wear out, leading to increased environmental damage. One way to improve this situation are woven fabrics made of polyester and cotton blends. It gives fabrics more tensile strength, so they can withstand multiplies commercial care procedures. However, the bed linens made of cotton-polyester blends are difficult to recycle, consuming significant amounts of energy and chemical solvents. The cotton upcycling is practically implausibly because of loss of fibres initial length causing decrease in tensile strength and ability to spinning^20^. Better solution is to use single fibre composition fabrics, but give an explicit attention to the quality of them, especially in case of fibres quality. To assess the durability of cotton textiles when subjected to repeated industrial washing, their tensile strength and dimension change are the primary factors taken into account, as stipulated by the standards OS 80-06 and OS 80-04^19,21^. The above mentioned requirements do not cover the effect of washing processes on changes in the molecular structure of cellulose fibers.

**Table 1:** Quality requirements for textile according to national standard OS 80-06:2015^19^.

Parameter	Value
Dimension change after 3 washes according to EN ISO 5077; EN ISO 6330, 9N procedure (white products), 6N (colour products)	Warp ± 3% Weft ± 3%
Tensile strength EN ISO 13934-1	Min. 400N

**Figure 1:**
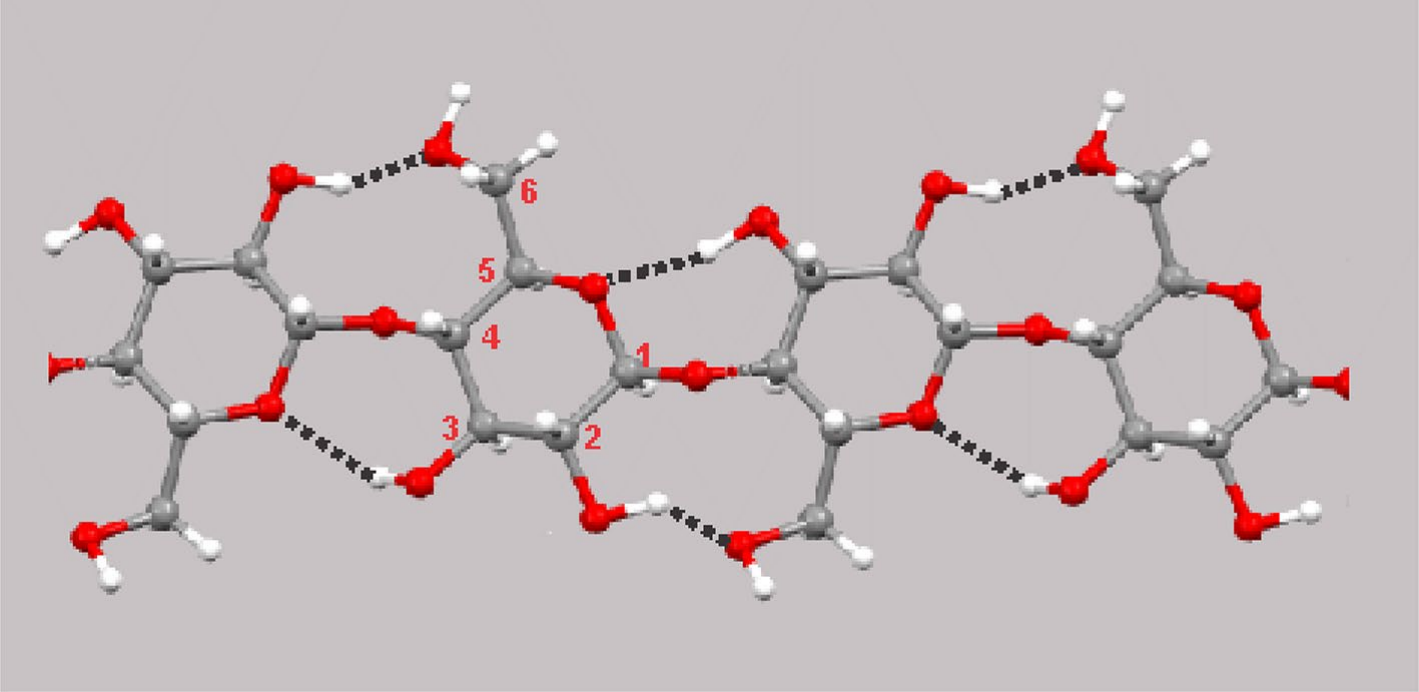
Chemical structure of cellulose^22^.

Cellulose, being the building block of the cotton fibers, is made up of glucose monomers (glucopyranose rings) linked by 1,4 β-glycosidic bonds (Fig. 1). The length of the chain (degree of polymerization of cellulose) and consequently the strength of cotton fibers are significantly influenced by the quantity of these bonds. In addition, the individual cellulose chains are linked to each other by hydrogen bonds occurring between atoms of –H and –OH groups.

To evaluate the degree of polymerization of the main material of cotton fibers, a method based on dissolving the fibers in a solution of cupri- ethylenediamine complex (CED) is used, as described in standard ISO 5351^23^. Methods for calculating the degree of polymerization vary in terms of the algorithm used and the type of cellulose solvent. When using the aforementioned cupri-ethylenediamine complex, calculation algorithms are available, as recommended in ISO 5351 and shown in Table 2.

**Table 2:** Method of degree of polymerization calculation for cellulose.

Algorithm source	Algorithm
SCAN CM 15^24^	${DP}^{0.905}-0.75\left[\eta \right]$
Evans^25^	${P}_{V}=1.65\left[\eta \right]$
Immergut^26^	$\left[\eta \right]=1.33\times {10}^{-4}{P}_{V}^{0.9}$
Gruber^27^	$\left[\eta \right]=1.7\times {10}^{-2}{DP}^{0.8}$
Da Silva Perez^28^	${DP}^{\mathrm{0,9}}=1.65\left[\eta \right]$

Designations used in Table 2: D, DPv—degree of polymerization, Pv, Mn—relative molecular mass of cellulose, [ƞ]—limiting viscosity number. In order to assess the strength parameters of fabrics and the detrimental effects of industrial laundering, ISO 2267^29^ specifies the use of a reference fabric and ISO 4312^30^ provides limits on the allowable changes in the parameters of this fabric following repeated maintenance processes (× 25, × 50). Damage to the reference fabric, resulting in non-compliance with the above-mentioned document requirements, means that the maintenance process is unsuitable for cotton fiber products, causing accelerated wear. Frequent industrial laundering processes can weaken cotton fabric, as the combination of mechanical and chemical factors can reduce its durability. The mechanical forces exerted during laundering, coupled with the redox reactions initiated by Cl- or H2O2-releasing compounds, contribute significantly to this phenomenon. According to ISO 4312, it is believed that the decline in strength of cotton fiber fabrics due to industrial washing processes has a linear correlation with the decrease in the limiting viscosity number [ƞ], which is determined by the method described in ISO 5351. This study aimed to investigate the correlation between the reduction in limiting viscosity numbers and strength for bedding materials composed of either 100% or 50% cotton fibers that have undergone numerous industrial washing cycles utilizing alkaline and oxidizing agents (including the presence of Cl-).

## Materials

Four bed linens and the reference fabric were selected for testing the resistance of products to repeated industrial laundering. These woven fabrics are dedicated to the packaging of bed linens, in particular for hotel and hospital use. Therefore, it was assumed that they are characterized by adequate durability life, in particular, resistance to repeated industrial laundering. The fabrics have comparable surface mass values and are available in two different weaves, with variations in the raw material composition, yarn type, and length of cotton fibers. The length of the fibers that were measured was obtained from the yarn; therefore, it does not accurately reflect the initial length of the cotton fibers at the start of the spinning process. The characteristics of the tested fabrics are shown in Table 3.

**Table 3:** Fabric characteristics.

Parameter	Fabric				
	1	2	3	4	5 (reference ISO 2267)
Raw material composition	100% cotton fibres	100% cotton fibres	50/50% cotton fibres/PES	50/50% cotton fibres/PES	100% cotton fibres
Weave pattern	Plain	Satin	Plain	Satin	Plain
Mass per unit area, g/m^2^ (ISO 3801)	148 ± 2	150 ± 8	145 ± 10	140 ± 7	165 ± 2
Linear mass, Tt (EN ISO 2060)					
Warp	30 tex	12 tex	20 tex	12 tex	25 tex
Weft	30 tex	12 tex	30 tex	12 tex	40 tex
Fibre length, mm					
Warp	10–20 mm	18–21 mm	35–40 mm	23–38 mm	20–25 mm
Weft	10–18 mm	22–30 mm	30–40 mm	28–35 mm	30–35 mm
Type of yarn	Rotor spun yarn	Ring spun yarn	Ring spun yarn	Ring spun yarn	Ring spun yarn
Threads density, 1/dm (EN 1049-2)					
Warp	250 ± 10	725 ± 5	300 ± 10	615 ± 10	250 ± 5
Weft	240 ± 10	385 ± 10	220 ± 10	270 ± 10	214 ± 10
Tensile strength, N (EN ISO 13934-1)					
Warp	420	600	400	600	710
Weft	400	400	400	400	670
Dimension change after 5 washings, % (EN ISO 5077; EN ISO 6330)					
Warp	− 5	− 3	− 5	− 3	− 3
Weft	− 5	− 3	− 5	− 3	− 3

The fabrics being the subject of the study in terms of tensile strength meet the basic requirements shown in Table 1. Two of the fabrics selected in plain weave have dimensions that exceed the allowed shrinkage limit, but this was only observed after undergoing five washing cycles.

## Test methods

The fabrics selected for the study were subjected to a series of preservation processes under industrial conditions (the laundry providing commercial laundry services). Each preservation process included washing and drying the fabric. The fabric samples were subjected to the following washing and drying multiples: 5, 10, 25, 50 and 100 processes. The Table 4 shows data on the machines and chemicals used during the washing process.

**Table 4:** Professional washing and drying conditions.

Process	Machine	Conditions	Chemical components Burnus Hychem
Washing	Lapauw 1600 drum washing machine (load 160 kg, steam heating) IPSO washing machine, loading max 20 kg	washing temperature 60 °C	Liquisan B (alkaline pre-wash detergent) Olisso Power (alkaline laundry detergent, with phosphates and fragrances, without soap, disinfecting properties) Trisanox (white activator, disinfectant properties)
Drying	Gas dryer Steam dryer	temperature 80 °C	

Additionally, fabric 1 was subjected to chlorine-based washing in the solution containing Cl^−^ under laboratory conditions. The washing conditions were determined by gathering information from the laundry regarding the standard concentration and composition of the washing solution containing sodium hypochlorite. As per the laundry statement, this process doesn’t harm the longevity of cotton products as it doesn’t cause any mechanical harm. Moreover, it effectively eliminates dirt and grey mold. Laundry has provided instructions for optimal washing conditions. For optimal results, it is advisable to apply a concentration of 13% sodium hypochlorite. The amount to use is 2 L per 250 L of washing bath. The washing process should last for 20 min at a temperature of 40 °C. In the laboratory, the washing process was carried out using the following parameters: a 4.3% solution of sodium hypochlorite, 6 mL for every 250 mL of solution, a washing time of 20 min, and a temperature of 40 °C. The washing bath and the sample were mixed during the entire washing process. The following times of washes were used: 5, 10, 25, 50 and 100—similarly as in the case of washings in industrial conditions.

After implementing the planned industrial washing procedures and using free chlorine during laboratory washing, the fabric samples went through further testing. The maximum force, which indicates tensile strength, was measured in accordance with EN ISO 13934-1. Limiting viscosity number was tested under the condition of standard ISO 5351. The viscosity limit number test was performed for yarns taken from fabrics with 100% cotton fibers, i.e. 1, 2 and 5, and fabric 4. In the case of fabric 4 (cotton/polyester blend), the polyester fibers were dissolved in phenol.

## Results

The tests yielded maximum force values for every fabric analysed, along with the limiting viscosity number and degree of cotton polymerization for cotton fabrics and fabric 4. The results of the tests include the values of both indicators determined after each interval of the number of industrial washes. Furthermore, the outcomes for fabric 1 following the specified amount of washes in an oxidizing solution with the presence of free chlorine are showcased.

### Durability

**Figure 2:**
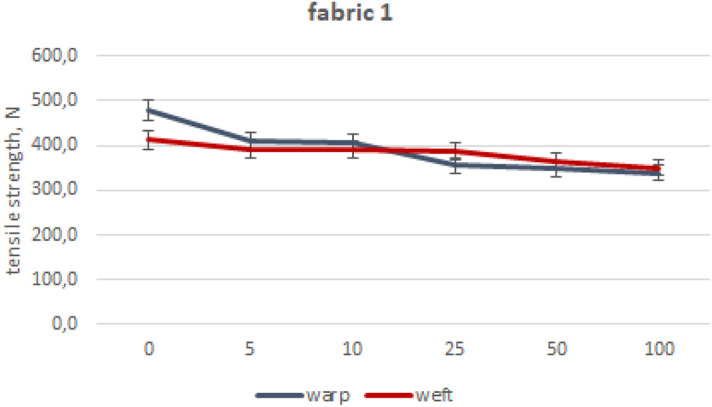
Woven fabric 1—changes of tensile strength.

**Figure 3:**
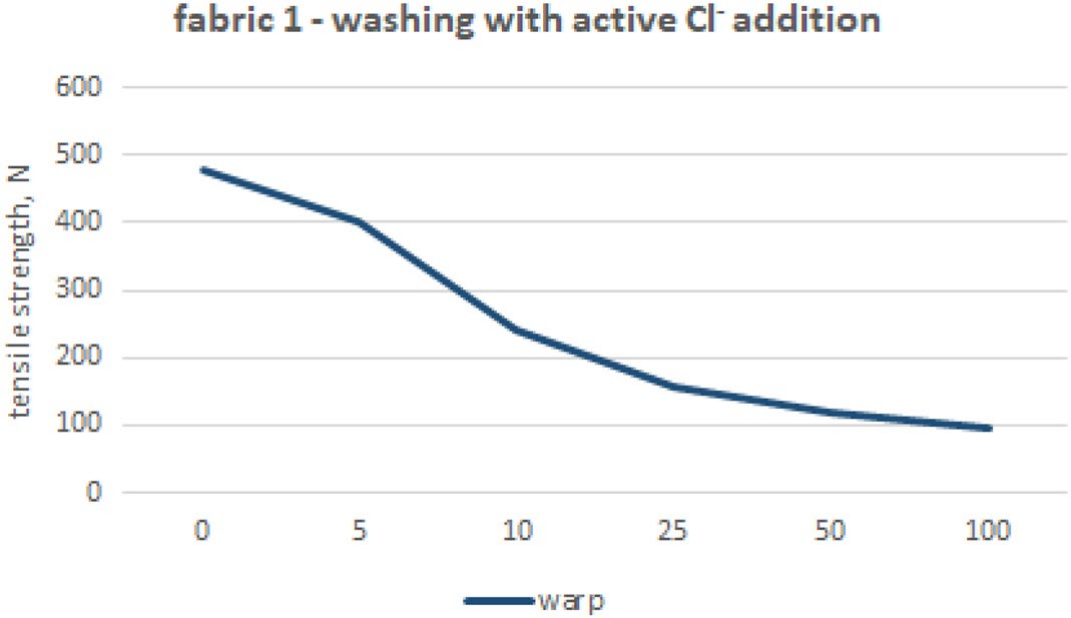
Woven fabric 1—changes of tensile strength (chlorine-based washings).

**Figure 4:**
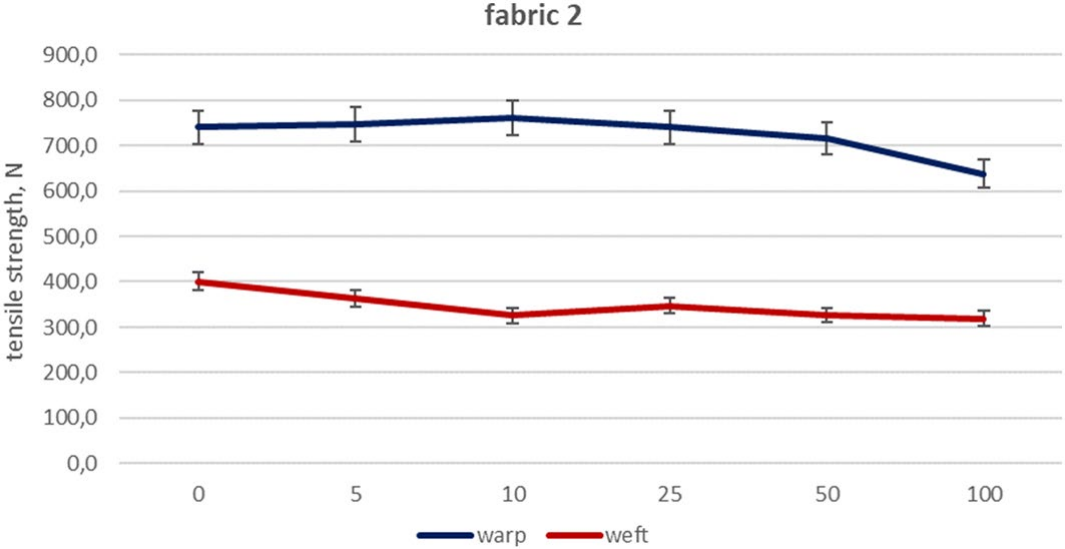
Woven fabric 2—changes of tensile strength.

**Figure 5:**
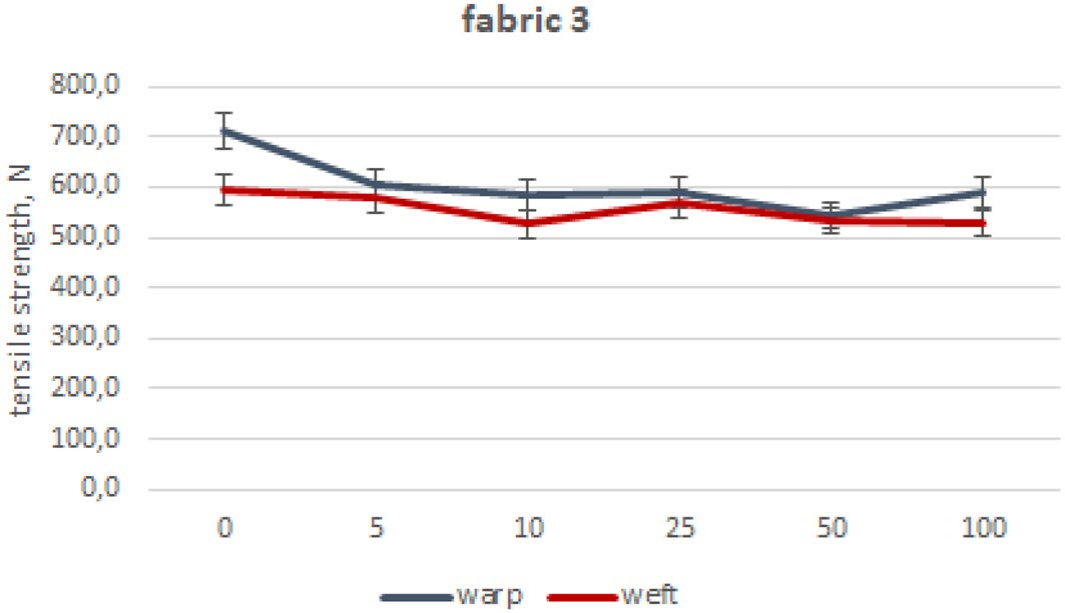
Woven fabric 3—changes of tensile strength.

**Figure 6:**
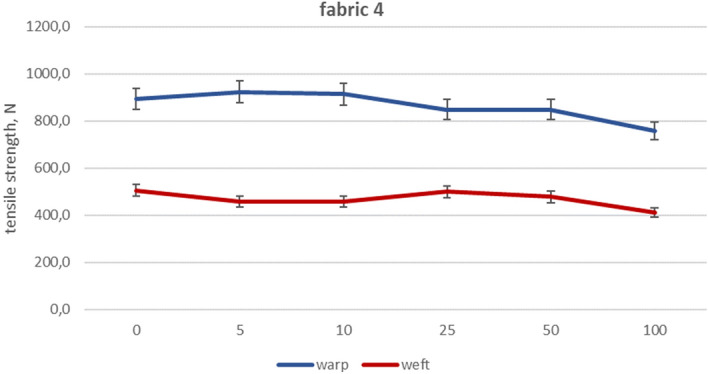
Woven fabric 4—changes of tensile strength.

**Figure 7:**
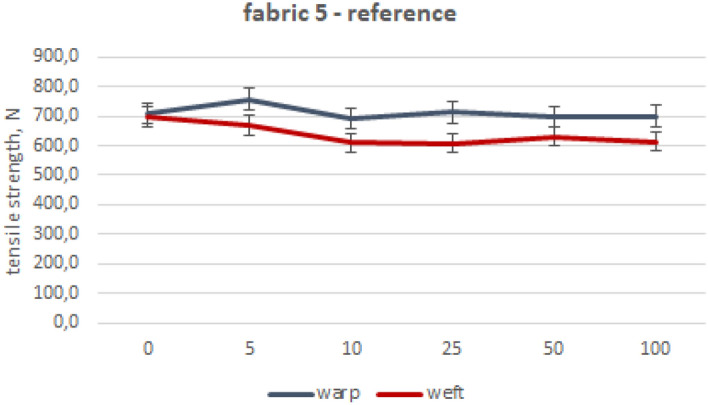
Woven fabric 5—changes of tensile strength.

The values of mean maximum force for particular fabrics subjected to industrial washing cycles are included in Figs. 2, 3, 4, 5, 6 and 7.

After conducting strength measurements on fabric 1, it can be inferred that industrial washing has a substantial impact on its tensile strength. Specifically, the warp direction experiences a significant decrease of 29%, while the weft direction decreases by 15% after undergoing 100 washing cycles. The majority of tensile strength loss occurred after the first five washing cycles, nearly 20% in warp direction. Going further, the strength drop plummeted after each set of washing cycles. The loss in fabric’s 1 tensile strength after industrial washing conditions is the most severe in comparison to the rest of tested fabrics. However, fabric 1 was made of open-end spun yarns, thicker than in other investigated fabrics. The fibre’s length is the shortest one as well. Fabric 1 has proven to be suitable for industrial washings multiple times, based on the OS 80-06^18^ standard, despite experiencing a significant decrease in tensile force values.

Figure 3 presents the decrease in the maximum strength of fabrics’ 1 warp threads, subjected to a chlorine-based washing process with free chlorine carried out in laboratory conditions. The industrial washings without chlorine-based components caused the highest reduction in tensile strength for the warp direction of Fabric 1, when compared to the weft direction and the other fabrics that were tested. Regarding chlorine-based treatment—16%, a drop in maximum force value after just five washes was observed. Then, it rises up to 50% after ten washes, coming to 80% after one hundred washes. These results could correspond to the yarn composition, especially the very short fiber length. On the other hand, the cotton fibers are especially vulnerable towards the free chlorine treatment, carried out in temperatures above 20 °C. After undergoing five wash cycles in the initial phase of fabric 1 destruction, there were only minimal visible indications of damage, such as a slight yellowing (corresponding to a level 4–5 color change on the grey scale according to EN ISO 105-A03:2019). As the number of washings processed, the yellowing intensified, reaching grade 2–3 at the end of tests. Significant negative changes were observed in the fabric’s hand and stiffness after subjecting samples to 100 washes in free chlorine solutions. The fabric even occasionally ruptured under typical handling operations, as determined through organoleptic assessment.

Taking under consideration tensile deterioration of fabric 2 (Fig. 4), the loss of maximum force value reaches 14% in warp direction and 7% for weft. The initial values of tensile strength are higher in comparison to the fabrics 1, which is connected with an increase in woven structure density, but with the fabric mass steady. On the other hand, satin weave enables to expose large parts of warp threads on the surface. Despite this, tensile strength did not drop significantly, reaching a good level, above 600 N after a hundred washings.

Fabrics 3 and 4 are made of polyester/cotton blended ring spun yarns. Blended fabrics exhibited a lesser reduction in maximum strength after 100 washes compared to pure cotton fabrics, with a decrease of up to 20%. The maximum force values obtained for fabric 3 plummeted to 17% in warp direction and 11% for weft direction, as shown in the Fig. 5. Slightly different tensile strength decrease took place for fabric 4 (Fig. 6)—for warp direction being 15% and 19% for weft. In the case of fabrics made of blended yarns, the drops in tensile strength are similar for both plain and satin weave patterns.

Reference fabric 5 exhibits the lowest decreases in both directions, only 1% in the direction of the warp and 12% for the weft, as was shown in Fig. 7. The observed huge difference between results obtained for other fabrics, especially fabric 1, could come from the yarn construction and cotton fibre length. Fabric 1 was manufactured using the rotor spun method for yarn production, which is notorious for causing fibre shortening, therefore it is likely to contain a greater proportion of short fibres. It could make fabric 1 less durable, in comparison to the fabric 5.

The analysis of the fabric’s strength during washing showed a decrease of no more than 30%. Despite having a comparable surface mass, fabric 3 woven in satin showed lower decrease compared to fabric 1 woven in plain weave because of the smaller number of thread arrangements. This is due to the different linear mass of yarn used in creating the fabrics. Moreover, fabric 1 was woven from other types of yarns, made of shorter cotton fibers. By evaluating the decay of fabric 1’s strength through industrial and laboratory washing processes using free chlorine, it is apparent that there is a considerable contrast in strength reduction as well as the appearance of the fabric. This is despite the laboratory process lacking an intensive mechanical interaction. The fabrics made of blended yarns are generally characterized by lower decreases in maximum strength after 100 washing processes (20%) compared to cotton fabrics (29%). The fabric 5 (reference) showed the lowest strength after 100 washing processes, especially when compared to fabric 1. Fabric 5 holds an advantage over the other fabric despite having the same weave pattern, similar thread counts and mass per unit area. This advantage comes from the difference in the manufacturing technique and the length of the fiber used in making the yarn. Taking under consideration the impact of woven structure on strength decline, cotton plain fabrics are more susceptible to losing their tensile strength than cotton satin fabrics. In the case of blended fabrics, both weave patterns show similar tensile strength loss. Reference fabric exhibits the best strength and durability after washings, despite their plain weaving pattern.

### Limiting viscosity number and degree of polymerization

The viscosity limit number tests were performed for fabrics 1, 2 and 5 made from 100% cotton fibres and fabric 4 made from blended yarns. In order to eliminate polyester fibres from the structure of warp and weft yarns, extraction with phenol solution was used. Furthermore, we also tested the impact of phenol exposure on cotton fibres by subjecting 100% cotton fibre yarns (from fabric 1) to the same phenol treatment used for the previous tests on other materials. The limiting viscosity number [ƞ] values obtained for the warp yarn of fabric 1 are 1682 (yarn without phenol exposure) and 1675 (yarn after phenol exposure), indicating that the difference between these values is no less than 1%. The absence of any impact of phenol exposure on cotton fibres from fabric 1 towards the limiting viscosity number of cellulose-forming fibres can be inferred. The results of the viscosity limit number test for the yarns of fabrics 1, 2, 4 and 5 are shown in Figures from 8 to 12.

**Figure 8:**
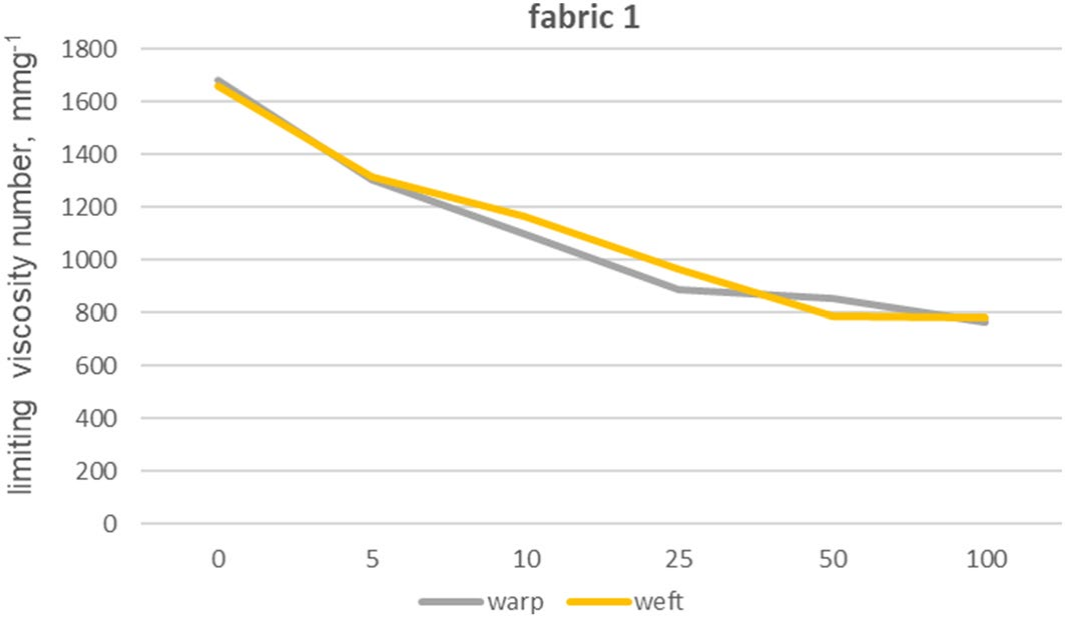
Limiting viscosity number after washings’—fabric 1.

**Figure 9:**
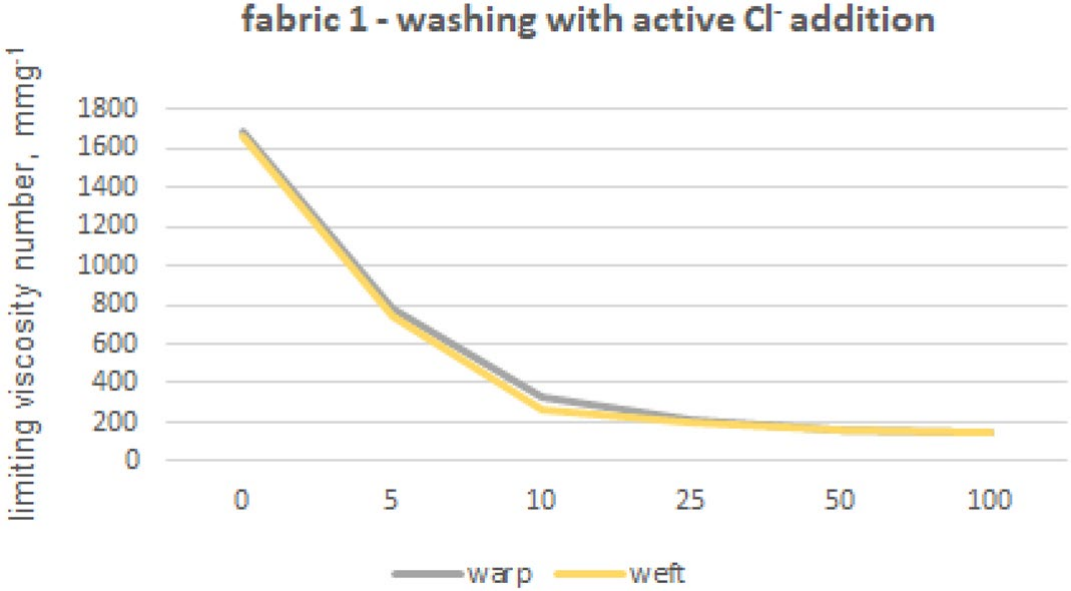
Limiting viscosity number after chlorine-based washings’—fabric 1.

**Figure 10:**
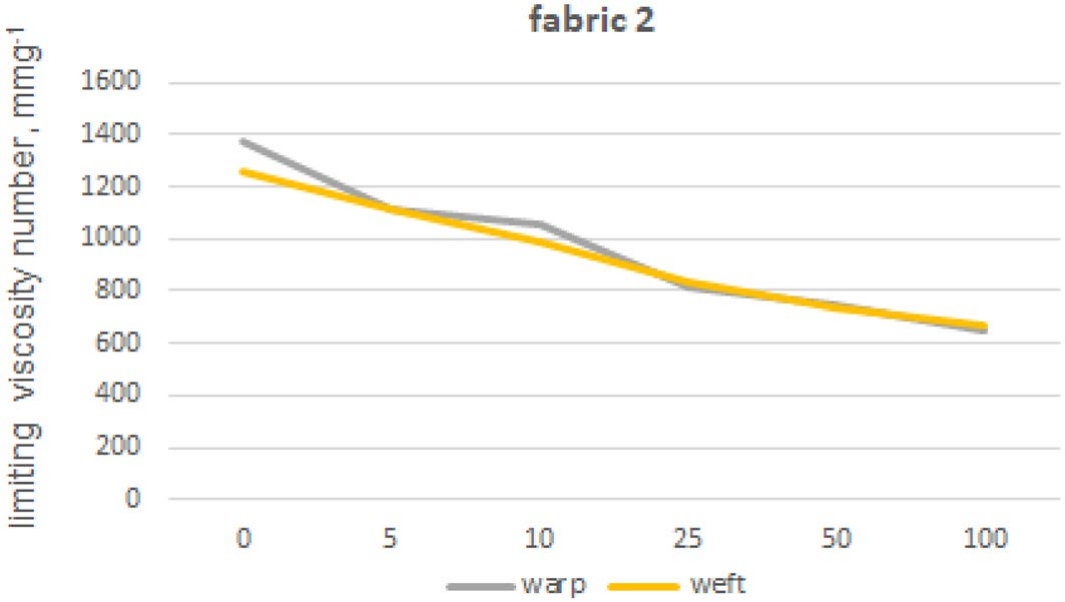
Limiting viscosity number after washings’—fabric 2.

Fabrics 1 (Fig. 8) and 2 (Fig. 10) have an initial high viscosity number that reduces by over fifty percent after undergoing 100 industrial washing processes. However, Fabric 1 experiences a rapid decrease in viscosity number, losing over 50% of its original value within the first five washes, and an additional 80% in the following five washes. It could be connected with shorter fiber content of the fabric 1, in comparison to the other fabrics. After being washed with a chlorine solution for a hundred cycles, fabric 1’s cotton fibers exhibited a significant decrease of over 90% in their viscosity number values, as demonstrated in Fig. 9. This caused the fibers to turn yellow and become fragile.

Fabrics 2 (Fig. 10) is characterized by the slightly less initial value of viscosity number in comparison to the fabric 1. This fabric loses half of this value after 100 industrial washing processes. In contrast, fabric 1 loses more than half of its initial viscosity number value after just the first five washings. The variation in the reduction gradient of limiting viscosity number between fabric 1 and 2 may be attributed to the predominance of yarn type and fiber length impact rather than weaving pattern.

**Figure 11:**
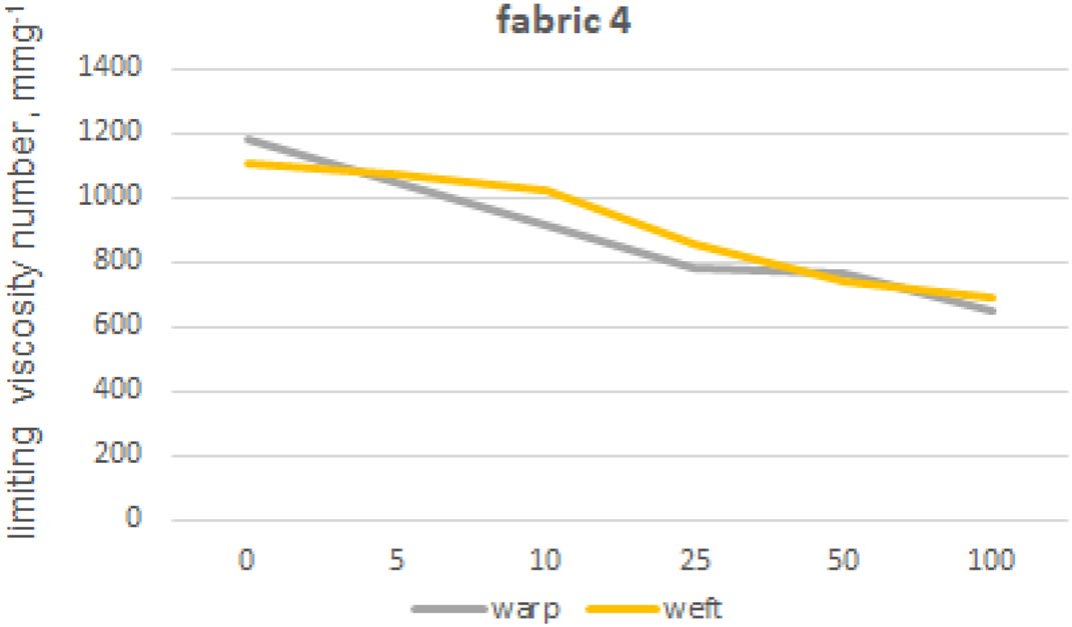
Limiting viscosity number after washings’—fabric 4.

Fabrics 4 (Fig. 11) present a lower initial value for the limiting viscosity number determined for cotton fibers compared to fabrics 1 and 2. Cotton fibers from fabric 4 loosed 45% of its value for warp direction and 37% for weft one after 100 industrial washing processes. There were the lowest drops of limiting viscosity numbers among all tested fabrics. However, the influence of cotton fibers degradation is less important in case of fabric 4, because the yarns are made of polyester/cotton blend. Even after a significant decrease in cotton fiber strength, the polyester component of yarn is able to carry out forces.

**Figure 12:**
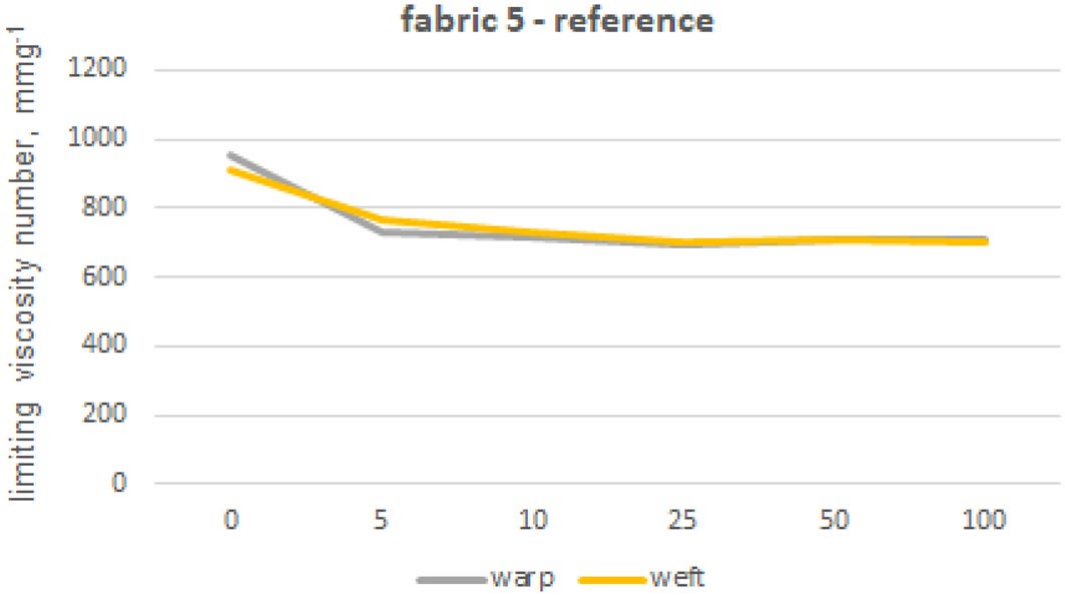
Limiting viscosity number after washings’—fabric 5.

The fabric 5 (Fig. 12) showed lower initial values of limiting viscosity number, with simultaneous lower decreases after washings, reaching only 23% its initial values. These phenomena could be related with cellulose molecular structure themselves. The initial limiting viscosity number may not only be associated with the precise number of β 1–4 glycosidic bond, but also with weaker hydrogen bonds that break down easily and deteriorate early on. Even after undergoing 100 industrial washing cycles, the cellulose chains remain highly intact as shown by a limiting viscosity number that exceeds 600. This phenomenon is consistent across various types of yarn, cotton fiber lengths and fabric structures tested. This confirms the previously observed strength drop—which does not exceed 30%—and serves as further proof of the durability of these fabrics.

The polymerization degree (DP) values of the tested fabrics were calculated using Algorithm (1) as described in standard SCAN CM 15:1999^24^. The results are shown in Table 5.

1 $${DP}^{\mathrm{0,905}}-0.75\left[\eta \right].$$

**Table 5:** Degree of polymerization of cellulose taken from fabrics.

Fabric	Mean value DP						
	Industrial washing cycles	0	5	10	25	50	100
1	Warp	2669	1304	1099	890	855	768
	Weft	2625	1312	1167	963	789	780
2	Warp	2136	1686	1588	1199	1084	935
	Weft	1936	1685	1488	1221	1074	972
4	Warp	1806	1583	1361	1147	1114	929
	Weft	1675	1626	1539	1269	1002	1073
5	Warp	1426	1065	1036	1007	1029	1029
	Weft	1359	1114	1061	1016	1026	1016

All samples tested after 100 washing processes achieved the polymerization degree about 1000 or slightly less, with initial values before washings at level 2600 ÷ 1400.

**Figure 13:**
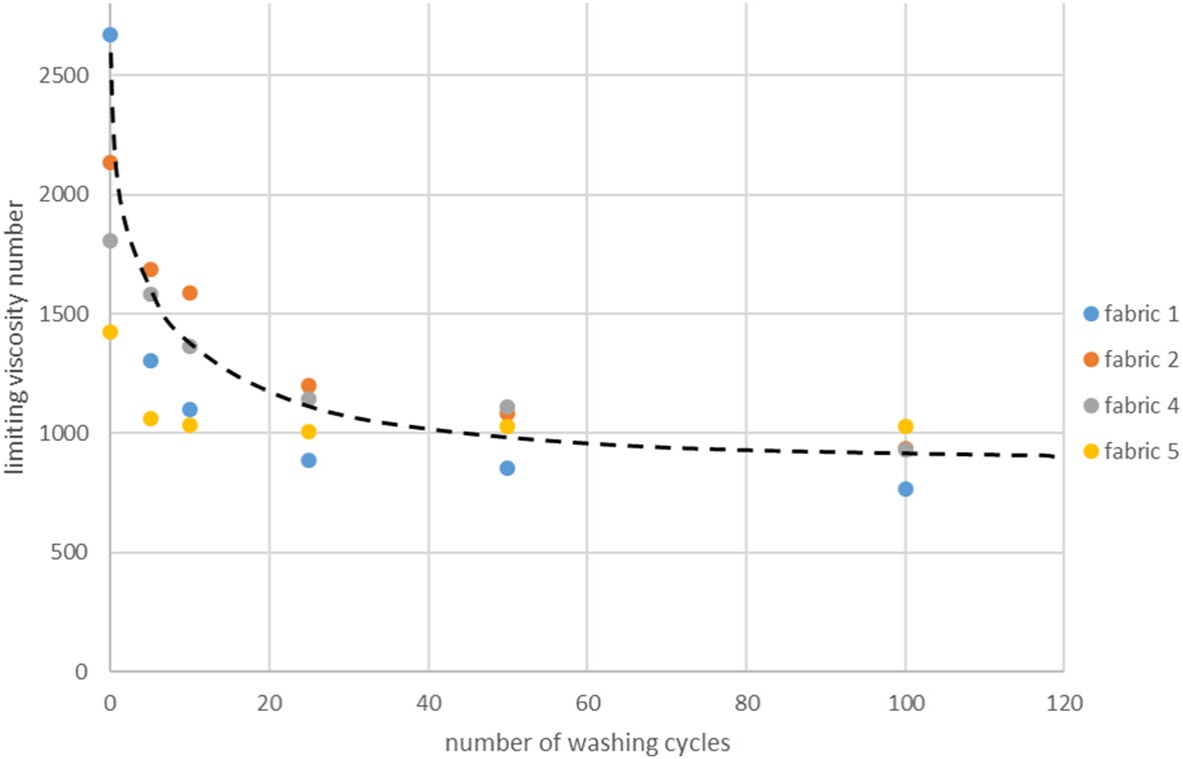
Approximation of n values in function of numbers of washings.

The logarithmic nature of the changes in the data obtained from measurements of the limiting viscosity number index can be easily observed by approximating it, as shown in Fig. 13 with a dotted line. It was found that together with the increase in the number of industrial washing processes, the decrease in the viscosity number is relatively small. The drop reaches its highest values at the beginning, with the number of washing processes up to 50. Similar to the function that calculates the degree of polymerization, the function that depicts the relationship between the limiting viscosity number and the degree of polymerization demonstrates an exponential nature. Therefore, the nature of changes in the limiting viscosity number value and the polymerization degree is very similar.

## Conclusions

By analysing the results of strength and viscosity tests conducted on cotton fabrics and blended fabrics, a visible difference in the trend of decreasing polymerization degree can be observed, which follows a logarithmic character, and fabric strength, which follows a linear pattern. As a result, it can be concluded that there is a notable disparity between the two. After undergoing 100 industrial washing cycles, the fabric experiences a more prominent reduction in polymerization degree, decreasing by 30–50%, in comparison to a decrease in maximum force of only 10–30%. Wherein, the fabrics 2, 4 and 5, which showed the smallest drops in the polymerization degree, also showed smaller drops in maximum force. Summarizing the analyses presented above, it can be stated that:

- The greatest decrease (up to 30%) in the cotton fabric strength was measured for plain woven fabric made of open-end spun yarns after 100 industrial washing procedures. At the same time, cotton fibers coming from this fabric are relatively short ones, but exhibits highest initial polymerization degree, equal to 2600. Meanwhile, the drop of polymerization degree observed for this fabric was the highest of all tested fabrics.
- In cotton fabrics, there was a correlation found whereby a lower initial degree of polymerization led to smaller decreases in strength after undergoing numerous industrial washing processes, even up to 100 cycles. It could be connected with inner molecular structure of cellulose, in particular with exact number and character of their bounds. This dependence also applies to cotton fibres taken from yarns of blended fabric.
- The correlations stated earlier are not relevant when laboratory washing using a chlorine-based solution was carried out. The reduction in the degree of polymerization varies from the reduction in strength, and it occurs rapidly within the first 10 washing cycles. After that, the decrease is only a few percentage points for every 25 or 50 washing processes. The fabric strength decreases more steadily, wherein the greatest decreases were also noted in up to 10 washing processes. The chlorine-based treatment causes severe damage to glycoside bounds in cellulose structure.
- Blended fabrics (50/50 cotton fibres/polyester fibres) have a lower strength loss after 100 washing processes than cotton fabrics, up to 20%. Blended fabrics have a higher level of durability in comparison to pure cotton fabrics due to the addition of polyester fibres into the yarn structure.

**Figure 14:**
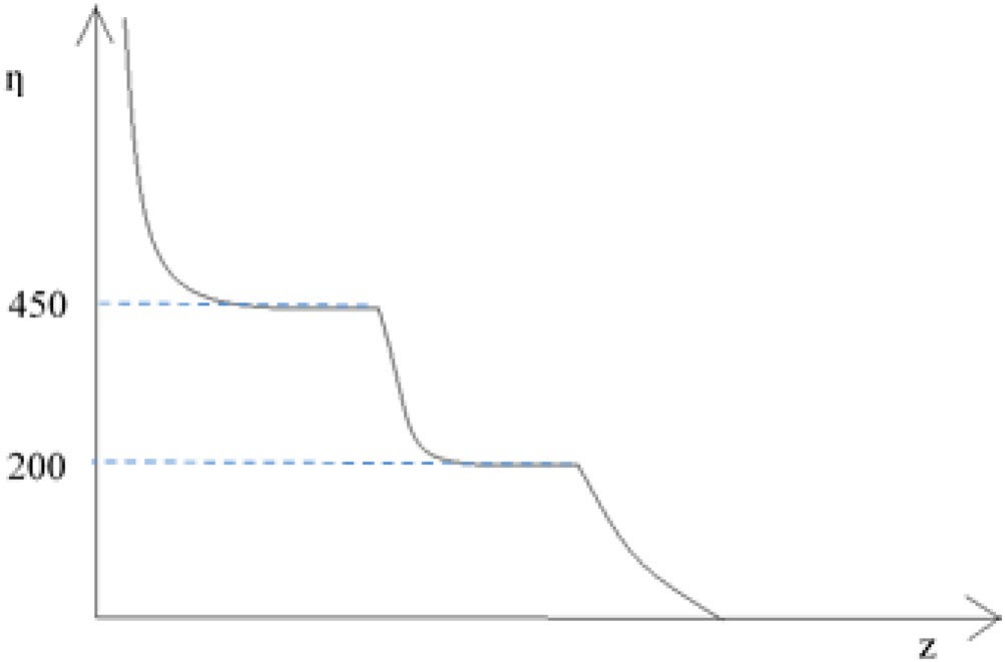
Limit viscosity number changes in progress of cellulose deterioration.

The previous tests and analyses reveal a noticeable reduction in the overall limiting viscosity number [ƞ] of cotton fibers and pulps after the treatment with an oxidizing bath containing chlorine ions. When cellulose first reaches the value of 450–500 mm g^−1^, then 200 mm g^−1^, two levels correspond to significant changes in the strength of the fabric (Fig. 14). During the initial phase of material structure alterations in these fibers, the [η] value experiences a Swift decline, irrespective of its initial magnitude, eventually stabilizing at a level of 450–500 mm g^−1^, which endures even in the face of prolonged cellulose degradation. If the limiting viscosity number is higher than 500, it means that the cotton fibers in the fabric are still strong and haven’t been damaged enough to cause a big decrease in its strength (less than 30% of the original value). Lowering the [ƞ] value will break down the β 1–4 glycosidic bond, resulting in a sudden and significant drop in the viscosity number to around 200 mm g^-1^. This level is maintained for a relatively long time despite the negative effects of the oxidizing agent on the cellulose. When the value of 200 mm g^−1^ is surpassed, the viscosity number limit drops quickly again until all the connections between the cellulose units are fully broken. Viscosity limit values that fall below 200 mm g^−1^ cause considerable fiber damage, ultimately leading to a significant reduction in the fabric’s strength, rendering it unusable.

## Summary

Based on the research and analysis conducted on the relationship between the mechanical strength and degree of polymerization of cotton fibres used in bed linen fabrics subjected to industrial washing conditions, it can be concluded that:

- cotton fabrics with limiting viscosity numbers over 600 should have mechanical strength at the level that ensures minimum durability,
- the initial value for unused fabric should be at least 900. Higher values do not increase the fabric’s resistance or prolong its durability after repeated industrial laundering,
- the reduction of [ƞ] value and its strength relies heavily on the upkeep procedure and the structure of the cleansing solution, particularly the inclusion of oxidizing agents like chlorine ions,
- Both blended fabrics containing a 50/50 cotton/polyester composition and cotton alone exhibit an outstanding resistance after undergoing a minimum of 100 washing cycles using chlorine-free detergents, with a strength loss that does not exceed 20–30%. Based on the values of limiting viscosity numbers acquired after 100 wash cycles and the logarithmic and multi-stage nature of β 1–4 glycosidic bond breakdown in cellulose chains, it is highly probable that the textiles will maintain their mechanical strength despite undergoing 200 or more washing procedures. By avoiding oxidants like chlorine ions during the maintenance process, it is possible to considerably enhance the number of subsequent maintenance processes. This measure can prevent rapid loss of strength and, consequently, deterioration of the fabric.

## Data Availability

The datasets used and/or analyzed during the current study available from the corresponding author on reasonable request.

## Abbreviations

DP: Degree of polymerization
P_V_: Relative molecular mass of cellulose
[ŋ]: Limit viscosity number
